# Determinants and dynamics of repeat unsafe abortion in Ghana—a mixed-method study at Tema General Hospital

**DOI:** 10.3389/fgwh.2026.1812634

**Published:** 2026-08-17

**Authors:** Kelvin Stefan Osafo, Sahadetu A. Mustapha-Sahabi, Derrick Ofosu Amoh, Pengming Sun

**Affiliations:** 1 College of Clinical Medicine for Obstetrics & Gynecology and Pediatrics, Fujian Medical University, Fuzhou, Fujian, China; 2 Fujian Clinical Research Center for Gynecologic Oncology, Fujian Maternity and Child Health Hospital, Fuzhou, China; 3 Fujian Key Laboratory of Women and Children’s Critical Diseases Research, Fujian Maternity and Child Health Hospital, Fuzhou, China; 4 Department of Population, Family and Reproductive Health, School of Public Health, Kwame Nkrumah University of Science and Technology, Kumasi, Ghana; 5 Overseas Education College, Fujian Medical University, Fuzhou, Fujian, China; 6 Department of Obstetrics and Gynecology, Tema General Hospital, Tema, Ghana

**Keywords:** contraception, Ghana, maternal health, post-abortion care, reproductive rights, unsafe abortion

## Abstract

**Background:**

Unsafe abortion remains a significant driver of maternal morbidity and mortality in Ghana. Despite progressive policies, many women continue to bypass safe clinical pathways. This study aims to identify the socio-demographic factors and qualitative dynamics associated with repeat unsafe abortion among women presenting with unsafe abortion complications at Tema General Hospital.

**Method:**

The study targeted women aged 15–49 years who were admitted for complications arising from unsafe abortions. A total of 68 women participated in the analysis, providing both quantitative and qualitative data. Quantitative data were analysed using Fisher's exact and Chi-square tests for bivariate associations, followed by Firth's penalised logistic regression to identify independent predictors. Simultaneously, qualitative semi-structured interviews explored the underlying motivations and personal dynamics behind termination decisions. All statistical analyses were performed in R, with a significance level of 0.05.

**Results:**

Age at sexual debut was the strongest independent predictor of repeat unsafe abortion, with over 14-fold higher odds among women who debuted at 15–19 versus later (AOR = 14.35, 95% CI: 2.30–170.29, *p* = 0.003), corroborated by qualitative accounts of early, unplanned pregnancies. Higher education showed a non-significant protective trend (AOR = 0.28, 95% CI: 0.05–1.06, *p* = 0.062), echoed in accounts of “recovery literacy” among educated women. The model explained 47% of the variance in repeat-abortion status (pseudo-R^2^ = 0.466). A widespread sense of “unpreparedness,” linked to financial instability and partner unreliability, led 70.6% of participants to rely on informal networks for at-home misoprostol, often unaware of complications.

**Conclusion:**

These findings underscore the urgent need for interventions to address the high rates of unsafe abortions in Ghana. There is a need for improved sexual education, promotion of contraceptive utilisation, and providing accurate information about safe and legal abortion options.

## Introduction

Maternal mortality remains a highly preventable but persistent global health concern. In 2023, approximately 260,000 women died during and following pregnancy and childbirth, with Sub-Saharan Africa accounting for 70% of these deaths (1). In Ghana specifically, the institutional maternal mortality ratio recorded at health facilities rose slightly to 101.68 per 100,000 live births in 2024, alongside a decline in skilled-delivery coverage to 55.3% (2). A significant contributor to this mortality is unsafe abortion (1, 3), which the World Health Organisation (WHO) defines as a pregnancy termination performed by individuals lacking necessary skills or in substandard environments (4). Globally, abortion contributes to approximately 8% of maternal deaths, with an estimated 45% of all abortions classified as unsafe (5, 6). The case-fatality rate is particularly alarming in regions such as Western and Central Africa, where it reaches an estimated 450 per 100,000 abortions (6). The decision to seek an abortion is deeply complex, heavily influenced by a woman's reproductive autonomy, socioeconomic status, and systemic barriers to healthcare access (7).

In Ghana, unsafe abortion is a leading cause of maternal mortality, with 15% of women of reproductive age reporting seeking unsafe abortions (8). Abortion rates in Ghana also vary from the northern to the coastal and middle regions, which are from the lowest to the highest, respectively, with approximately 200,000 abortions occurring in the country (9). Abortion laws in Ghana are among the most liberal in Sub-Saharan Africa, permitting termination on grounds that include rape, incest, fetal abnormality, and risk to the mother's physical or mental health (10), with clinical guidelines that have themselves been described as a potential model for other low- and middle-income countries (11). Despite the Ghanaian government's initiatives to enhance abortion laws, barriers remain, including cultural stigma and insufficient implementation of existing policies (10, 12). Many health care providers are reluctant to perform abortions due to societal perceptions, leading to a reliance on unsafe methods (12). Consequently, many women continue to bypass safe clinical pathways, relying instead on unsafe, informal methods.

Despite increasing evidence on unsafe abortion in Ghana, important knowledge gaps remain. Previous studies have largely focused on estimating the prevalence of unsafe abortion or identifying broad sociodemographic correlates using mainly either quantitative or qualitative methods (8, 13). Consequently, limited evidence exists regarding the factors associated with *repeat* unsafe abortion and the complex individual, interpersonal, and societal mechanisms that influence women's decisions to seek unsafe abortion services. Furthermore, few studies have integrated quantitative and qualitative approaches to simultaneously identify predictors and explore the contextual drivers underlying unsafe abortion practices.

To our knowledge, this is among the first studies in Ghana to use a concurrent mixed-methods design to identify quantitative predictors of repeat, specifically, unsafe abortion while simultaneously situating those predictors within women's own accounts of why they returned to unsafe methods. Understanding these factors influencing repeat unsafe abortion is crucial for developing targeted interventions across the nation. By identifying determinants and engaging the community through education and dialogue, the study also aims to contribute to reducing the incidence of unsafe abortions and improving reproductive health outcomes in line with one of the targets of Sustainable Development Goal 3, which seeks to lower maternal mortality rates (14).

## Methods

### Study design, population, and sampling

A cross-sectional study was conducted on women who attended the Tema General Hospital for abortion-related complications from December 2022 to May 2023. The study population included women of reproductive age admitted to Tema General Hospital. The inclusion criteria were women aged 15–49 admitted for abortion-related complications who provided informed consent, while the exclusion criteria were women who declined participation or were unable to provide consent. A total of 70 eligible patients were enrolled during the data collection period. After data collection, the questionnaires were rigorously validated and cleaned. Two participants were later excluded from the analysis due to significant missing data or unresolved inconsistencies in their responses.

### Data collection

Data were collected using two primary instruments: structured questionnaires and semi-structured in-depth interviews. All data collection was conducted with assistance from trained midwives and researchers. The midwives received a week-long training session on conducting field interviews. They were informed about the objectives and nature of the study. As the interview was conducted mainly in the Akan language (Twi), translations of common questions, concepts, and terms were agreed upon in advance to ensure consistency. The study tools explored personal experiences, decision-making processes, perceived barriers to safe abortion services, and community attitudes, collecting oral responses from participants, which were transcribed in real time. The tools captured the following:

1. Socio-demographic Variables: Age, education, employment, marital status, religion, and residence.
2. Knowledge Assessment: Awareness of abortion laws, safe abortion services, and contraceptive use.
3. Behavioural Factors: Reasons for unsafe abortion, methods used, providers, and decision-making dynamics.
4. Clinical Data: Abortion history, complications, and post-abortion care experiences.

Given the sensitive nature of abortion disclosure, interviews were conducted in private, one-on-one settings without family members or other patients present, and participants were reminded they could decline any question or stop at any time. Participants were identified with letters and numbers to maintain anonymity: PP11 (participant participant number 11). A purposive sampling approach was utilised, with all 68 survey respondents completing semi-structured interviews. Because this study utilised an integrated mixed-methods design, the final sample size was determined by the complete quantitative cohort rather than thematic data saturation, ensuring that each participant's quantitative data was directly contextualised by their qualitative narrative.

### Robust

Pre-testing was conducted at La General Hospital to refine the questionnaire. The pre-test helped researchers to modify questions for clarity. The pre-test also helped ensure that the collected data answered the research questions. The research team engaged in prolonged interactions with participants to ensure an in-depth understanding of emerging findings. Data transcription and coding were conducted by the research team to ensure that participants’ experiences and views were accurately reported.

### Data analysis

Quantitative data were analysed using R software (version 4.5.1). Descriptive statistics, including frequencies and percentages, were used to summarise the participants’ socio-demographic characteristics. Bivariate associations between potential determinants and the frequency of unsafe abortion (categorised as single vs repeat) and other associations (e.g., education and abortion knowledge) were assessed using the Chi-square test or Fisher's exact test, where appropriate (Fisher's exact test was used when expected cell frequencies were less than 5). The effect size (Cramer's V) to assess the strength of the association between variables.

To identify independent predictors of repeat unsafe abortion while accounting for the small sample size and potential issues of monotone likelihood (separation), a multivariable analysis was conducted using Firth's Penalised Logistic Regression. Variables with *p* < 0.25 in bivariate analysis and those considered epidemiologically important were entered into the multivariable model. The final model included age of sexual debut, current age as a confounder, and education level. Model fit was evaluated using the likelihood ratio test and Nagelkerke's pseudo-R^2^. Statistical significance was set at *p* < 0.05, and results were reported as Adjusted Odds Ratios (AOR) with 95% Confidence Intervals (CI). Qualitative responses were thematically analysed to contextualise the quantitative findings, as previously described by Atakro and colleagues (8). Qualitative data from the semi-structured interviews were transcribed verbatim and analysed using a thematic analysis framework. To ensure qualitative rigor, two independent researchers coded the transcripts. An initial coding framework was developed based on the study objectives and emerging concepts from the first set of transcripts. The coders independently applied this framework to the data, categorising responses into overarching themes such as “unpreparedness,” “partner unreliability,” and “stigma.” Inter-coder agreement was assessed through iterative discussions; any discrepancies in code application or theme generation were resolved through consensus, with a third senior researcher consulted to finalise the thematic structure. These qualitative themes were then triangulated with the quantitative findings to contextualise the statistical predictors of repeat unsafe abortion.

## Results

A total of 70 patients were initially eligible; after excluding those with inconsistent responses, the final analysis was performed on a complete, validated dataset of 68 participants. The socio-demographic characteristics of the patients are shown in Table 1. The study found that 69.1% of the included patients were in the 20–29 age group, making it the largest group; 27.9% were over 30 years old, and only 2.9% were teenagers (17–19). Most patients had some level of formal education: 41.2% had attended only secondary education (senior high school), 20.6% had completed tertiary education, and 23.5% had completed junior high school. However, 4.4% had no formal education. Most patients were either single or in consensual relationships (35.3% each), followed by those still married (26.5%), with only two patients separated or divorced. The study also found that patients with 1–2 parities (children) had the highest number of abortions (66), while those with 3 or more children and no children had fewer. Most patients identified themselves as Christians, with only seven identifying as Muslims.

**Table 1:** Socio-demographic characteristics of patients.

Characteristic	*N* = 68*^1^*
Age Group	
17–19	2 (2.94)
20–29	47 (69.12)
30+	19 (27.94)
Level of Education	
No Formal Education	3 (4.40)
Primary	7 (10.30)
Junior High School	16 (23.50)
Senior High School	28 (41.20)
Tertiary	14 (20.60)
Employment Status	
Unemployed	28 (41.20)
Employed	40 (58.80)
Marital Status	
Single	24 (35.30)
Divorced/Separated	2 (2.90)
Consensual Relationship	24 (35.30)
Married	18 (26.50)
Intended Children	
1–2	2 (2.90)
3–4	61 (89.70)
5+	4 (5.90)
No response	1 (1.50)
Parity	
Nulliparous	12 (17.65)
1–2	40 (58.82)
≥3	16 (23.53)
Area of Residence	
Rural	32 (47.10)
Urban	36 (52.90)
Religion	
Islam	4 (5.90)
Christianity	64 (94.10)
*1* *=* *n (%)*	

This table shows the socio-demographic profile of the 68 patients participating in the study. All data are presented as frequencies (n) followed by percentages (%).

Table 2 shows that most of the patients’ partners were aged 30–39 (42.6%) and 20–29 (33.8%), followed by those aged 40 + years, with the fewest aged less than 20 years. The patients’ responses showed that many partners had either senior high or tertiary education (41.2% each), while only 2.9% had no formal education. The results also showed that 91.2% of the respondents’ partners were employed and 8.8% were unemployed, with approximately 57.4% living in urban areas and 42.6% in rural areas.

**Table 2:** Partner demographic characteristics.

Characteristic	*N* = 68 *^1^*
Age Group	
<20	1 (1.5)
20–29	23 (33.8)
30–39	29 (42.6)
40+	15 (22.1)
Level of Education	
No Formal Education	2 (2.9)
Junior High School	10 (14.7)
Senior High School	28 (41.2)
Tertiary	28 (41.2)
Employment Status	
Unemployed	6 (8.8)
Employed	62 (91.2)
Area of Residence	
Rural	29 (42.6)
Urban	39 (57.4)
*1* = n (%)	

This table presents the demographic characteristics of the partners of the 68 participants. These statistics provide context on the home environment and the support systems around the patients. All data are presented as frequencies (n) followed by percentages (%).

The study further explored why women chose to end their pregnancy and how many abortions they had. The results show that this was the first abortion for 34(50%) patients and the second abortion for 30 patients. Only four patients had had three or four abortions, as shown in Table 3. Our qualitative results provided a deeper understanding of Table 3, revealing the complex individual, interpersonal, and societal factors. At the individual level, the findings showed that the decision to get an abortion was mainly due to personal feelings of unpreparedness to have a child at the time.

**Table 3:** Unsafe abortion counts and reasons.

Variable	*N* = 68 *^1^*
Abortion count	
1	34 (50.0)
2	30 (44.1)
3	3 (4.4)
4	1 (1.5)
Reason	
Educational pursuits	12 (17.6)
Financial constraints	1 (1.5)
Health concerns	4 (5.9)
Lack of knowledge on safe services	1 (1.5)
Personal goals	2 (2.9)
Relationship issues	6 (8.8)
Social stigma	11 (16.2)
Unplanned	31 (45.6)
*1* = n (%)	

Table 3 summarizes the frequency of unsafe abortions and the reasons reported by the 68 participants. Half of the respondents (50.0%) experienced one abortion, while 44.1% had two. The main reason for seeking an unsafe abortion was that the pregnancy was unplanned (45.6). Other key factors included the desire to continue education (17.6%) and concerns about social stigma (16.2). Additional reasons noted were relationship problems (8.8%) and health concerns (5.9). Data are shown as counts (n) and percentages (%).

“No, I’m okay with the children that I have. Even if I become financially stable, I’m all right.” pp. 8

“I was so disappointed because I told myself that after this first pregnancy, I was not going to be pregnant any time soon. So, when I found out I was pregnant, I was devastated and had mixed emotions… I just decided that it's the best choice for me to terminate until I’m financially, emotionally, and mentally ready for a second child.” pp. 18

“Because I wasn't ready for that child… I think also because I have a small baby, I felt that she needs more attention, because she is one year old, so I felt that a baby plus a young child, I don't think I will be able to balance that.” pp. 39

Additionally, 14 patients (20.6%) cited educational or occupational pursuits (personal goals) in their responses as the reason for termination (Table 3).

“I admitted to my partner that I wasn't prepared since I wanted to finish my education and that being pregnant would cause me to be late.” pp. 4

“What would I do if I were to obtain employment?” pp. 66

Another reason that stood out is health-related.

“I wanted to lose weight before my next pregnancy since my relatives kept scaring me that my pregnancy would be complicated if I were heavy, so I decided to end the pregnancy when I got pregnant.” pp. 10

Interpersonally, partner dynamics shaped these decisions as much as the pregnancies themselves — and not always in ways that track partners’ own circumstances. Table 2 shows that most partners were employed (91.2%) and had at least a senior high school education (82.4%), yet this economic stability did not reliably translate into relational support. Participants in the study reported a wide range of experiences regarding their partners’ involvement in the decision to terminate a pregnancy. Some women had partners who were actively involved in the decision-making process, leading to a joint decision to terminate the pregnancy.

“I told him I wasn't prepared. While he was not yet ready, he concurred as well.”

However, many others faced significant instability and lacked support from their partners. Only 8.8% of women named “relationship issues” as their primary reason (Table 3). Over half of the women in the qualitative sample cited concerns about their relationship or fear of being a single mother as a reason for termination. This included not being in a committed relationship, a partner's unwillingness to take accountability for the pregnancy, or the relationship suffering after the pregnancy was disclosed. Specific relationship issues included physical abuse, infidelity, unreliability, and immaturity. Many women felt let down because their partners disputed paternity, cut off contact, or stated that they did not want the child. These factors made it more difficult for participants to accept their pregnancies and have faith in their partners.

The qualitative results also showed that societal factors influencing the abortion decision arose from the family, the place of worship, and the community, which was the main reason for 11 patients (16.2%) (Table 3).

“The only idea they have is that killing is wrong; thus, they oppose termination. (Because) You are unable (unallowed) to do something, so when the consequences arise, you choose to face them head-on.” pp. 37

Most participants stated that although these were widely held beliefs that seemed to influence their opinions on abortion, they neither affected their decisions nor dissuaded them from terminating their pregnancies. However, several individuals noted that there was stigma and condemnation surrounding abortion in their society. Some of the patients had a perception that having an abortion is quite normal and widespread in their communities, hence influencing their decision and reducing how they felt about the associated stigma.

“It's common, but like if you're doing things alone, since other people do it alone, just like I did mine alone. I'm the only one who knew. I'll therefore say that it is typical. Because we hear rumours and some people choose to view things for themselves despite not knowing, I believe they are frequent.” pp. 11

The study also aimed to determine where they terminated their pregnancies, the methods used, and the sources of those methods, as shown in Table 4. The combination of individual urgency, unreliable partner support, and privatised stigma, as seen from the qualitative results, pushed most women toward the same practical solution: home-based, informally sourced abortion. The results reveal that most participants (91.18%) opted for home abortions, while the rest (8.82%) sought assistance from private hospitals/clinics or maternity wards. The results further outline the specific methods used to terminate pregnancies. Notably, approximately 58.8% of respondents relied solely on oral medications. Among those who could recall, the most used oral drug was misoprostol, also known as “Cytotec.” Additionally, about 4.4% of the patients reported using abortion-inducing insertion medicines, while around 13.2% and 2.9% mentioned herbal remedies and homemade concoctions, respectively. The study also found that pharmacists, partners, or friends were often the primary providers/source of abortion procedures.

**Table 4:** Places, providers, and methods of unsafe abortions.

Variable	*N* = 68 *^1^*
Place of Abortion	
Home	62 (91.18)
Private Hospital/Clinic	5 (7.35)
Private Maternity Home	1 (1.47)
Abortion Method Used	
Herbs Only	9 (13.24)
Home-made Concoctions Only	2 (2.94)
Insertion Medicines Only	3 (4.41)
Oral Medication Only	40 (58.82)
*Combination 1 (H & HC)	1 (1.47)
*Combination 2 (H, HC, & IM)	3 (4.41)
*Combination 3 (HC & IM)	2 (2.94)
*Combination 4 (OM, H, & HC)	1 (1.47)
*Combination 5 (OM & IM)	7 (10.29)
Provider/Source of Abortion Method	
Friend	39 (57.35)
Partner	14 (20.59)
Pharmacist	11 (16.18)
Nurse/Midwife	2 (2.94)
Relative	1 (1.47)
Self-thought	1 (1.47)
Awareness of Method's Complications	
No	48 (70.60)
Yes	20 (29.40)
*1* *=* n (%)	

This table shows the logistics and risks linked to unsafe abortions.

*H=Herbs; HC=Home-made Concoctions, IM=Insertion Medicines; OM=Oral Medication.

Bivariate analysis (Table 5) initially showed that age at sexual debut (*p* < 0.001; Supplementary Figure S1) and education level (*p* = 0.048) were significantly linked to the frequency of unsafe abortions. To identify independent predictors while accounting for confounding factors such as current age, Firth's penalised logistic regression was conducted. The overall multivariable model was statistically significant (Likelihood Ratio *χ*^2^(5) = 28.08, *p* < 0.001). The model's explanatory power was assessed using Nagelkerke's pseudo-R^2^, which indicated that the included variables accounted for approximately 47% of the variance in repeat abortion outcomes. The model indicated that age at sexual debut is the strongest independent predictor of repeat unsafe abortion. Women who started sexual activity between ages 15–19 were 14.35 times more likely to have a repeat procedure compared to those who initiated earlier (AOR = 14.35; 95% CI: 2.30–170.29; *p* = 0.003). Additionally, a notable trend was observed with education; women with higher education were about 72% less likely to experience a repeat abortion compared to those with only basic education (AOR = 0.28; 95% CI: 0.05–1.06), though this finding was not statistically significant (*p* = 0.062). Current age did not significantly influence the likelihood of repeat procedures after accounting for sexual debut.

**Table 5:** Bivariate and multivariable analysis of factors associated with repeat unsafe abortion*.

Variable	Single Abortion n (%)	Multiple Abortion n (%)	Bivariate *p*-value	Adjusted OR (95% CI)	Adjusted *p*-value
Age at sexual debut			<0.001		
10–14	6 (17.6)	1 (2.9)		1.00 (Ref)	
15–19	12 (35.3)	31 (91.2)		14.35 (2.30–170.29)	0.003
20+	16 (47.1)	2 (5.9)		0.47 (0.05–6.11)	0.527
Current Age			0.326		
20–29 (Ref)	22 (64.7)	25 (73.5)		1.00 (Ref)	
17–19	2 (5.9)	0 (0)		0.19 (0.00–3.19)	0.262
30+	10 (29.4)	9 (26.5)		1.84 (0.44–9.40)	0.408
Education			0.048		
Basic (Ref)	14 (41.2)	12 (35.3)		1.00 (Ref)	
Higher	20 (58.8)	22 (64.7)		0.28 (0.05–1.06)	0.062
Employment			0.805		
Unemployed	15 (44.1)	13 (38.2)		–	–
Employed	19 (55.9)	21 (61.8)		–	–
Marital Status			0.135		
Single	14 (41.2)	10 (29.4)		–	–
Divorced/Separated	2 (5.9)	0		–	–
Consensual Union	8 (23.5)	16 (47.1)		–	–
Married	10 (29.4)	8 (23.5)		–	–
Intended Children			0.542		
1–2	1 (2.9)	1 (2.9)		–	–
3–4	29 (85.3)	32 (94.1)		–	–
5+	3 (8.8)	1 (2.9)		–	–
No response	1 (2.9)	0		–	–
Parity			0.420		
0	8 (23.5)	4 (11.8)		–	–
1–2	18 (52.9)	22 (64.7)		–	–
3+	8 (23.5)	8 (23.5)		–	–
Area of Residence			0.808		
Rural	17 (50)	15 (44.1)		–	–
Urban	17 (50)	19 (55.9)		–	–
Religion			1.000		
Christianity	32 (94.1)	32 (94.1)		–	–
Islam	2 (5.9)	2 (5.9)		–	–

Table 5 examines factors associated with repeat unsafe abortions by comparing patients who had a single abortion to those with multiple abortions.

*Bivariate *p*-values were obtained through Fisher's Exact or Chi-Square tests, while Adjusted Odds Ratios (AOR) were calculated using Firth's Penalized Logistic Regression to account for the small sample size (N=68).

We also aimed to assess the level of knowledge about abortion among patients, as shown in Table 6. The results indicate that approximately 68% were aware of the abortion law, while the remaining did not know about it. Patients were also asked about their familiarity with various methods and tools used for safe pregnancy termination. Only 39.7% of the patients were aware of safe abortion services. Additionally, the patients were queried about their knowledge of contraceptives. Most patients (94.1%) were aware of contraceptives. Our qualitative findings showed that most patients had a lot of false beliefs about contraception. Several of them claimed to know about contraception, yet the majority held misinformed beliefs about it. Although many respondents were aware of CuT (an IUD), they believed it may result in perforation and death, which suggests serious misinformation. Few people were aware of OC tablets or thought they would have health effects. Some patients’ partners utilised barrier contraception, however, inconsistently, because they felt embarrassed to buy condoms from stores.

**Table 6:** Knowledge on abortion.

Characteristic	*N* = 68
Perception about Abortion	
Expensive	23 (33.8)
Safe	12 (17.6)
Both	33 (48.5)
Knowledge on Abortion Law	
No	46 (67.6)
Yes	22 (32.4)
Knowledge on Safe Abortion Services	
No	41 (60.3)
Yes	27 (39.7)
Knowledge about Abortion Instruments	
No	38 (55.9)
Yes	30 (44.1)
Knowledge about Contraceptives	
No	4 (5.9)
Yes	64 (94.1)
*1* *=* n (%)	

This table summarizes the participants' knowledge and perceptions regarding abortion and reproductive health. Perceptions of the procedure were mixed; 48.5% saw abortion as both expensive and safe, while 33.8% considered it strictly expensive, and 17.6% viewed it strictly as safe. These results highlight a gap between awareness of contraceptives and knowledge of legal or clinical aspects of abortion.

**Figure 1:**
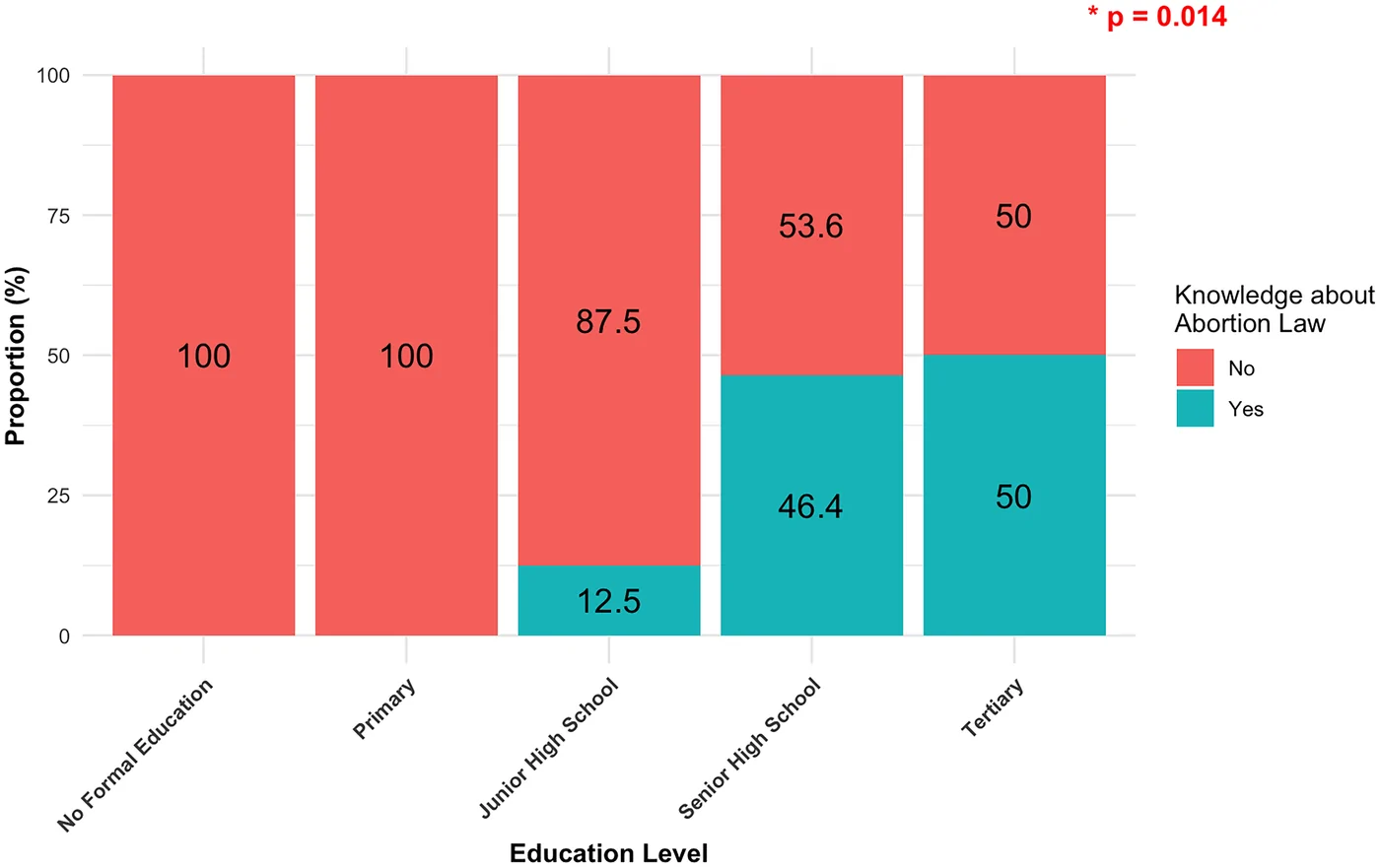
Association between educational level and knowledge about abortion law. Knowledge of abortion was not high at all levels of education. There is an increasing trend in the knowledge of abortion, beginning from Junior high school to tertiary education. Cramer's V showed a moderate association between education and knowledge about the abortion law (*p* = 0.014, Cramer's V = 0.423).

We further analysed the association between education level and awareness of abortion law using the chi-square test. The results indicated an association (*p* = 0.014), while Cramer's V = 0.423 indicated a moderate association between them. However, awareness of the abortion law was not high across all educational levels, as shown in Figure 1.

**Figure 2:**
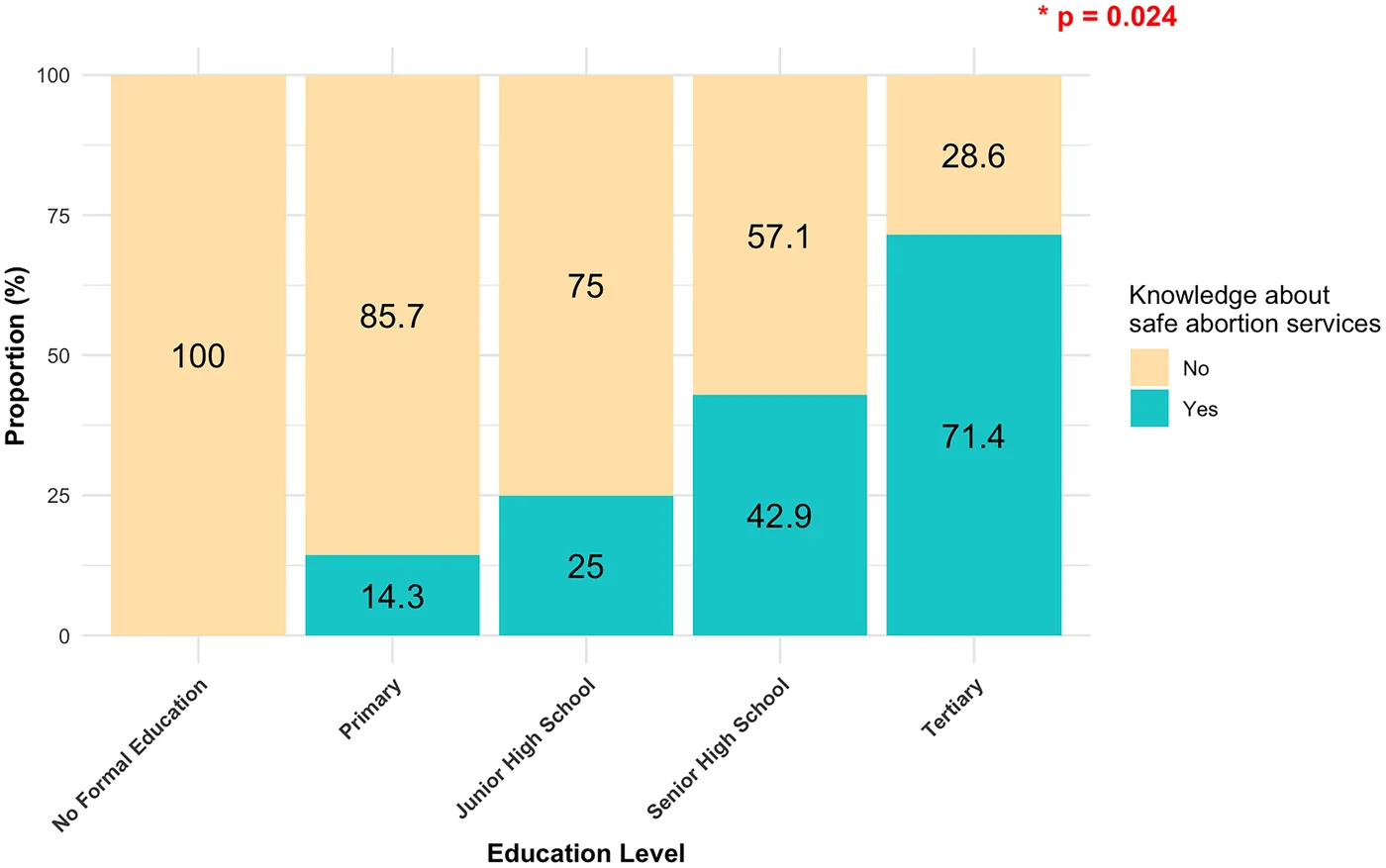
Association between educational level and knowledge about safe abortion services. There was an increasing trend in the knowledge of safe abortion services, even among those with only primary education. At the tertiary level, most of them were aware of safe abortion services. Analysis showed a moderate association between education level and knowledge about safe abortion services (*p* = 0.024, Cramer's V = 0.408).

Additionally, we analysed the association between educational levels and knowledge about safe abortion services. The results also showed a moderate association (*p* = 0.024, Cramer's V = 0.408). The findings additionally indicated that those with a tertiary education had greater knowledge about safe abortion services (Figure 2). Finally, our results showed no association between educational level and contraceptive knowledge (*p* = 0.23; Supplementary Figure S2). At every level, there was high awareness of contraceptives.

## Discussion

Unsafe abortion remains a critical public health challenge in Ghana, particularly among women of reproductive age (15–49 years), as evidenced by the findings of our study. This study highlights the complex interplay of sociodemographic, socioeconomic, and cultural factors that drive women to resort to unsafe abortion practices, undermining progress toward Sustainable Development Goals (SDGs) 3 (Good Health and Well-being) and 5 (Gender Equality). The results reveal that a predominant majority (69.1%) were aged 20–29, and most had attained at least a secondary education, yet a significant portion (41.2%) were unemployed. This suggests that young, educated, but often economically vulnerable women are disproportionately represented among those seeking unsafe abortions, reflecting gendered economic disparities (SDG 5.a) (15). These findings are quite common in LMICs settings where early reproductive risk-taking predicted adverse outcomes (16); socioeconomic vulnerability drove unsafe methods (17); younger, poorer, less-educated women faced higher unsafe-abortion risk (18).

A critical finding in our study is the strong association between the age of sexual debut and the number of abortions. Patients who began sexual intercourse at the ages of 15–19 had the highest number of repeated abortions. This suggests that sexual intercourse at an early age may be a risk factor for subsequent repeated unwanted pregnancies and subsequent repeated unsafe abortions later in life. This observation aligns with findings from a larger longitudinal study in Angola, reinforcing the external validity of the association between early sexual debut and repeat abortion despite our smaller cohort (16).

To further understand the magnitude of this risk, our multivariable analysis provided a good statistical anchor. The model revealed that an early sexual debut (15–19 years) was associated with a higher likelihood of repeat unsafe abortion by over 14-fold (AOR = 14.35, *p* = 0.003). Although the association was statistically significant, the wide confidence interval suggests limited precision due to the small sample size. Our qualitative data further explicitly suggest that this risk is due to early sexual debut initiating a compounding cycle of ‘unpreparedness,’ as early unplanned pregnancies conflict directly with a young woman's educational goals amid financial instability. This pattern echoes a national analysis of the same 2017 Ghana Maternal Health Survey, in which women citing education or career advancement as their abortion reason had 6.5 times higher odds of obtaining a least-safe abortion than those with a legally recognised reason (95% CI: 2.37–17.87) (19). Furthermore, the model's Nagelkerke pseudo-R^2^ of 0.466 suggests that nearly half (47%) of the variance in repeat abortion occurrences is explained by just three factors: the timing of sexual debut, education level, and current age. This moderate-to-high explanatory power validates the study's focus on these socio-demographic drivers. While the remaining 53% of variance likely involves the complex interpersonal dynamics found in our interviews—such as partner pressure, financial instability, and the intense social stigma surrounding premarital pregnancy—our model highlights that the ‘structural’ pillars of age and education may be the most significant levers for intervention. In practical terms, this points to a specific, actionable lever: delaying sexual debut even by a few years through comprehensive, age-appropriate sexuality education starting before adolescence, paired with early contraceptive counselling, could meaningfully reduce a woman's lifetime risk of repeat unsafe abortion in this setting.

These structural vulnerabilities are reflected in the specific reasons women gave for seeking termination. Our descriptive results (Table 3) showed the main reason cited for abortion was unplanned pregnancy, with the qualitative data from the interviews backing this finding, with personal unpreparedness supported by financial and emotional instability, and personal and educational goals. Moreover, interpersonal factors, particularly partner unreliability, were significant contributors to patients’ final decisions. This finding aligns with Chemlal and Russo's finding that socioeconomic safety was a reason for unsafe abortions (20). Furthermore, unemployed women and those in single or consensual unions faced higher chances of unsafe abortion, emphasising the intersection of poverty and limited reproductive autonomy, as also demonstrated by Rizvi and colleagues (17). Addressing these issues requires multicultural interventions, including youth-friendly contraceptive services (SDG 3.8) and programs that challenge gender norms (SDG 5.1) (14, 15).

Another important finding is that most of the abortion procedures were performed at home using oral medications, with misoprostol being the most commonly used. This is consistent with Chemlal and Russo's study (20). Pharmacists, friends, and partners were the main sources of these methods, highlighting an informal network that facilitates unsafe practices, as previously highlighted by Keogh and colleagues (21). This may be common in LMICs, where a systematic review across multiple countries found informal social networks frequently serve as care pathways when formal services are stigmatised or inaccessible — similar to our pharmacist-and-friend network (22). In our study, reliance on this network was 94.1% (Table 4), despite a relatively liberal law, indicating that women's trust in the formal system is the main barrier, not legal restrictions. This reliance likely reflects more than ignorance; women prefer pharmacists and peers for speed, privacy, and non-judgment. Pharmacists have become informal abortion providers, dispensing misoprostol without counselling, dosage guidance, or follow-up. These practices align with global patterns where restrictive abortion laws or poor awareness correlate with higher maternal morbidity and mortality (23). This underscores the urgent need for policy reforms and community education to align with SDG 3.7 (universal access to sexual and reproductive healthcare) and SDG 4.7 (education on gender equality and reproductive rights) (14, 24). Retraining pharmacists could be a low-cost, high-impact way to complement efforts to expand formal comprehensive abortion care access.

Alarmingly, 70.6% of participants were unaware of the potential complications associated with their chosen method. We believe a key determinant of repeated unsafe abortion is the lack of awareness regarding safe abortion. Our bivariate analysis suggests that low levels of education (awareness) led to higher levels of repeated abortion. Interestingly, while the bivariate analysis showed education was a factor, the multivariable model suggests its effect is partially mediated by the timing of sexual debut. The protective trend of higher education (AOR = 0.28), although statistically insignificant, suggests that while educated women are not immune to unsafe abortion, as evidenced by our qualitative interviews, suggesting that higher education may confer a form of higher ‘recovery literacy’ that reduces the likelihood of a second unsafe event compared to their less-educated peers (13). Furthermore, knowledge regarding abortion law and safe services was generally poor across all educational levels, though a moderate association was found, indicating that higher education slightly improved awareness. This correlates with the findings of a Swedish study, which stated that low levels of education are a factor in having incorrect knowledge of the abortion law (25). Also, a Ghanaian study indicated that people with knowledge of the abortion laws and secondary and tertiary education were less likely to use unsafe methods for abortion (13). In contrast, knowledge of contraceptives was nearly widespread (94.1%), yet our qualitative data demonstrated extensive misinformation that likely hinders effective use.

The study also highlights the unsaid role of healthcare systems in unsafe abortions. Despite Ghana's progressive abortion policy, inadequate service provision and provider biases deter women from accessing safe care (12). This aligns with more recent evidence that knowledge gaps extend to facility-level staff themselves, not just patients (26). Strengthening health infrastructure (SDG 3.c) and training providers in comprehensive abortion care (CAC) are critical steps in increasing access to safe abortion services, as concluded in an Ethiopian study (14, 27). This may reduce complications and mortality associated with abortion (28). Furthermore, integrating post-abortion family planning services could reduce repeat unintended pregnancies, hence aligning with SDG 3.7 targets (14, 29, 30).

Our study has limitations. First, this was a single-centre study with a small sample size (*n* = 68) using purposive, hospital-based sampling; this design, combined with the small sample size, limits the statistical power and the generalisability of our findings to women outside Tema General Hospital or to those who never reach facility-based care at all. Second, selection bias may have been introduced by excluding participants with inconsistent responses, and by the inherent exclusion of women whose unsafe abortions did not require hospital admission, or who avoided hospital care altogether — both groups may differ systematically from our sample. Third, our reliance on self-reported abortion histories raises the possibility of recall or social-desirability bias, as women may underreport prior abortions given the associated stigma. Future studies employing larger, multi-site, and, where feasible, community-based (rather than exclusively facility-based) samples are needed to validate and extend these findings.

## Conclusion, alignment with Sustainable Development Goals (SDGs), and recommendations

This study showed that repeat unsafe abortion was driven by early sexual debut, and, to a lesser extent, lower education – both operating through a compounding cycle of unpreparedness and reliance on unsupervised, informal-network methods, despite Ghana's comparatively liberal legal grounds. Closing this gap will require the coordinated, multi-level action set out to curb the prevalence of unsafe abortion. This study demonstrates the urgent need for improved sexual education, targeted correction of contraceptive misinformation through school-based programs and community sensitisation. It also highlights the need for health provider training and enhanced access to safe reproductive healthcare services to reduce reliance on unsafe methods.

### Alignment with SDG 3: good health and well-being

The study underscores the urgent need to reduce maternal morbidity and mortality linked to unsafe abortions (SDG Target 3.1). Complications from unsafe procedures accounted for 74% of hospital admissions, highlighting gaps in healthcare access and education. Strengthening comprehensive abortion care (CAC) services, integrating post-abortion care (PAC) with family planning, and expanding healthcare provider training are essential to mitigate risks. Addressing these barriers aligns with SDG 3.7, which advocates for universal access to sexual and reproductive healthcare services.

### Alignment with SDG 5: gender equality

Unsafe abortions disproportionately affect women due to gendered socioeconomic disparities and limited autonomy over reproductive choices (SDG Target 5.6). The study found that partner influence, stigma, and financial dependence often coerced women into unsafe practices. Empowering women through education, legal awareness, and economic opportunities can reduce reliance on unsafe methods. Furthermore, combating cultural and religious stigma may be critical to achieving gender equity in healthcare decision-making.

### Linkage with SDG 4 (quality education)

Misconception of contraception despite high knowledge of contraceptives and resorting to unsafe methods calls for enhanced sexual education in schools.

## Recommendations

Short-term:

1. Pharmacist and provider training: Comprehensive Abortion Care (CAC) training for nurses, doctors, counsellors, and pharmacists, targeting informal misoprostol-dispensing and provider bias.
2. Community awareness campaign: Ministry of Health-run multi-platform outreach (radio, social media, community and religious leaders) clarifying Ghana's abortion law and correcting the specific misconceptions.

Medium-term:

1. Decentralise CAC services: Ministry of Health extends comprehensive abortion care to primary and community facilities, cutting travel and stigma barriers, with District Health Directorates and NGOs.
2. Link to economic empowerment: Ministry of Gender, Children and Social Protection connects post-abortion and family planning services to existing empowerment programmes for young women, with NGO and District Assembly partners.

Long-term:

1. Curriculum reform: Ghana Education Service/Ministry of Education integrate mandatory, age-appropriate sexuality education covering abortion law and contraception into national curricula, with MoH and UNFPA.
2. Monitoring and evaluation: Track repeat-vs-first-time PAC presentations, provider knowledge scores pre/post-training, and population awareness of the law and safe services via periodic surveys — baseline at launch, follow-up at 24 months.

## Data Availability

The datasets presented in this article are not readily available because protecting and maintaining participants’ anonymity and confidentiality, and preventing their feelings from becoming public, remain a priority. Data will only be available on a reasonable request. Requests to access the datasets should be directed to Kelvin Stefan Osafo, osafokelvinstefan@fjmu.edu.com.
