# Supplementary material for: Determinants and dynamics of repeat unsafe abortion in Ghana—a mixed-method study at Tema General Hospital
Source: Front Glob Womens Health. 2026 Aug 17;7:1812634. doi: 10.3389/fgwh.2026.1812634 (PMC13526947; doi:10.3389/fgwh.2026.1812634)
Supplement: Supplementary file 1 [file Datasheet1.docx]

Supplementary Figures

Supplementary Figure 1. Association between the age of sexual debut and the number of abortions.

Patients who were aged 15-19 had the highest number of abortions, 91.2% had more than one abortion, and 35.3% of them had a single abortion. Those who were aged 20 and above were the second highest, with only 5.9 having more than one abortion, and 47.1 having a single abortion. Among those who were aged 10-14, 2.9% had more than one abortion, and 17.6% had a single abortion. The analysis revealed a strong link between the age of first sexual intercourse and the number of abortions (p<0.001, Cramer’s V=0.58).

Supplementary Figure 2. Association between educational level and knowledge about contraception.

There was no association. There is a high awareness of contraceptives at all levels of education.
